# Supplementary material for: Identification of photoperiod-induced specific miRNAs in the adrenal glands of Sunite sheep (Ovis aries)
Source: Front Vet Sci. 2022 Jul 22;9:888207. doi: 10.3389/fvets.2022.888207 (PMC9354845; doi:10.3389/fvets.2022.888207)
Supplement: Supplementary file 1 [file Data_Sheet_1.ZIP › Supplementary file/miRDeep2 file.zip!/miRDeep2 file/Novel_Structure/LP42A1/Novel_155.pdf]

Provisional ID	:	NC_019458.2_1477
Score total	:	61.8
Score for star read(s)	:	3.9
Score for read counts	:	54.2
Score for mfe	:	2.2
Score for randfold	:	1.6
Score for cons. seed	:	
Total read count	:	118
Mature read count	:	117
Loop read count	:	0
Star read count	:	1

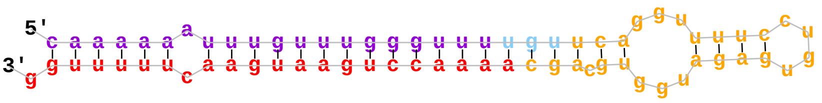

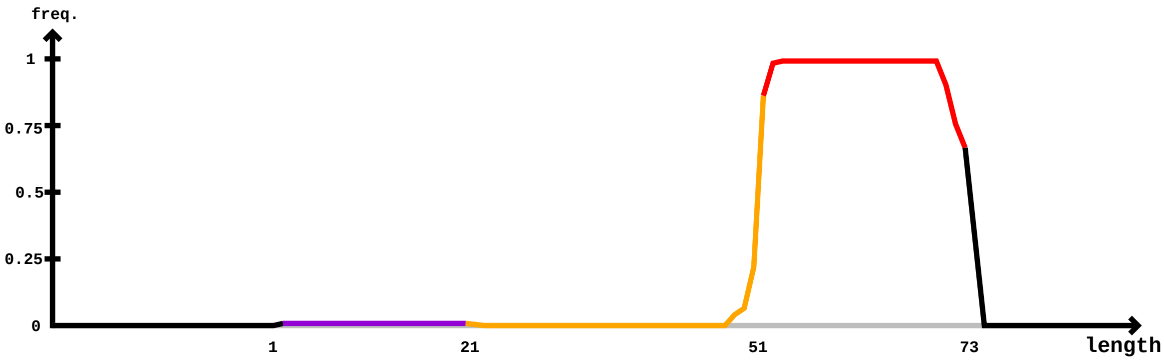

Star Mature

```
5'- uuaucagaaguauuggguuggccaaaaaauuuguuuggguuuuguucagguuuuccugugagauggugcagcaaaaccugaaugaacuuuuuggccagcccaauguuauuu  -3'  obs
    uuaucagaaguauuggguuggccaaaaaauuuguuuggguuuuguucagguuuuccugugagauggugcagcaaaaccugaaugaacuuuuuggccagcccaauguuauuu       exp
    ..........((((((((((((((((((.(((((((((((((((((((...((((....))))...)).))))))))))))))))).))))))))))))))))))......  reads  mm  sample
    ......................caaaaaGuuuguuuggguuu.....................................................................  1      1   seq
    .....................................................................agAaaaaccugaaugaacuuuu....................  5      1   seq
    ......................................................................gcaaaaccugaaugaacuuuu....................  1      0   seq
    ......................................................................gcaaaaccugaaugaacuuuuu...................  1      0   seq
    ......................................................................gGaaaaccugaaugaacuuuuu...................  1      1   seq
    .......................................................................Aaaaaccugaaugaacuuuu....................  4      1   seq
    .......................................................................Aaaaaccugaaugaacuuuuu...................  15     1   seq
    ........................................................................aaaacUugaaugaacuuuu....................  1      1   seq
    ........................................................................aaaaccAgaaugaacuuuuug..................  5      1   seq
    ........................................................................aaaaccugaaCgaacuuuuug..................  2      1   seq
    ........................................................................aaaaccugaaugaacuuuuuA..................  2      1   seq
    ........................................................................aaaacUugaaugaacuuuuug..................  1      1   seq
    ........................................................................aaaCccugaaugaacuuuuug..................  1      1   seq
    ........................................................................Gaaaccugaaugaacuuuuugg.................  2      1   seq
    ........................................................................aaaCccugaaugaacuuuuugg.................  2      1   seq
    ........................................................................aaaaccugaaugaacuuuuugA.................  12     1   seq
    ........................................................................aaaaccugaaugaacuuuuugg.................  40     0   seq
    ........................................................................aaaaAcugaaugaacuuuuugg.................  1      1   seq
    ........................................................................aaaaccugaaugaacuuuuuggA................  8      1   seq
    ........................................................................aaaaccugaaugaacuuuuuggG................  2      1   seq
    .........................................................................aaaccugaaugaacuuuuu...................  1      0   seq
    .........................................................................aaaccugaaugaacuuuuugU.................  1      1   seq
    .........................................................................aaaccugaaugaacuuuuugg.................  5      0   seq
    .........................................................................aaaccCgaaugaacuuuuuggc................  7      1   seq
    .........................................................................aaaccugaGugaacuuuuuggc................  1      1   seq
    ..........................................................................aaccugaaugaacuuuuugg.................  1      0   seq
```
